# The Metrizer: an innovative device for achieving virtual hepatic biopsies

**DOI:** 10.1186/1746-1596-8-S1-S17

**Published:** 2013-09-30

**Authors:** Carlo Russo, Barbara Franceschini, Sonia Di Biccari, Stefano Musardo, Guido Bosticco, Nicola Dioguardi

**Affiliations:** 1 Laboratory of Quantitative Medicine, Istituto Clinico Humanitas IRCCS, Rozzano, Milan, Italy; 2 University of Pavia, Pavia, Italy

## Background

The last few years have brought rapid growth in the number of Virtual Microscopes (VM) and the promotion of digital histology. In this digital era, it is natural to assist in the transportation of histology imaging into computer technology support. We have also assisted in a quick and precise race towards improved technology capable of acquiring detailed digital images which realistically report the original slide for easy consultation [1,2].

The advanced technology herein proposed aims at being a sophisticated imaging archive. Digitally scanning slides however, does not give additional information to the vision of tissue structures, even with high resolution or improved colour and image precision. We can now say that high enlargement microscopic observation can only give same details to structures visible to the eye directly through the microscope. This means that when VMs are used in a correct manner they are capable of giving significant progress to highlighting, not quantifying, a critical field in applying technology specific to computerised measuring. The potential to improve precision and objectivity of measurements can be achieved with additional technical equipment. The aim of this study is to present a machine invented with a calculation potential to facilitate the work of the observer in a medical practice, not only in terms of easy retrieval of images but also as an instrument for the automatic analysis of digital histology. The “Metrizer” aims at supplying precise and objective descriptions and measurements of the specimen under observation. These descriptions do not substitute the pathologist but they should assist him/her to assert with objectivity and safety the entity of the observed pathology [3-8].

## Material and methods

**Figure 1:**
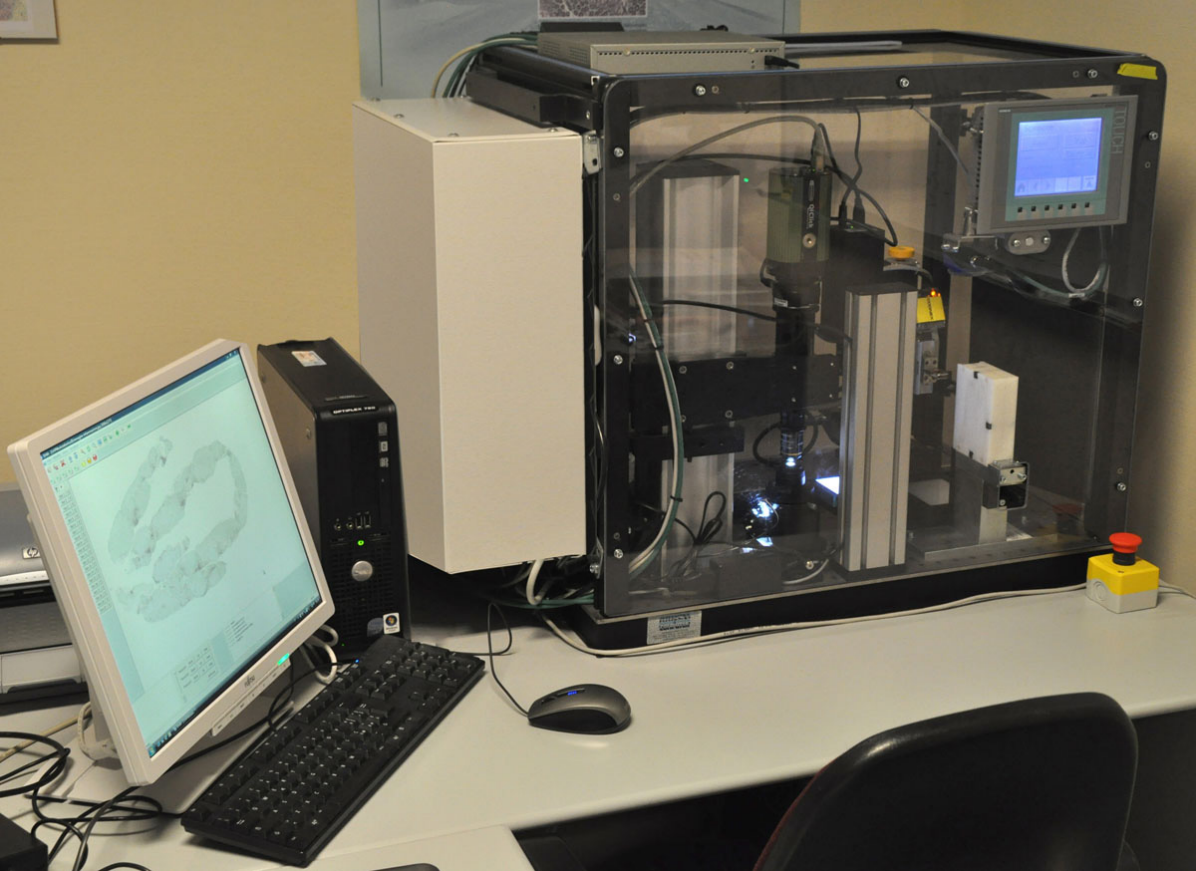
**The Metrizer** Prototypical version of the Metrizer, used in this work and for presentation purposes

The “Metrizer” (Figure 1) is an automatic, compact machine composed of a lens for microscopic observation, digital cameras, a movement device and a computer complete with software to control machine movement and image analysis. With these components the machine facilitates consultation of histological preparations (virtual microscope - slide scanner function) and supplies objective numeric data regarding the state of the tissue harbouring diverse diseases without causing fatigue or human error.

Using the Slide Scanner function it enables to automatically digitalise the entire histological slide in high definition and makes it easily accessible in the digital archive.

**Figure 2:**
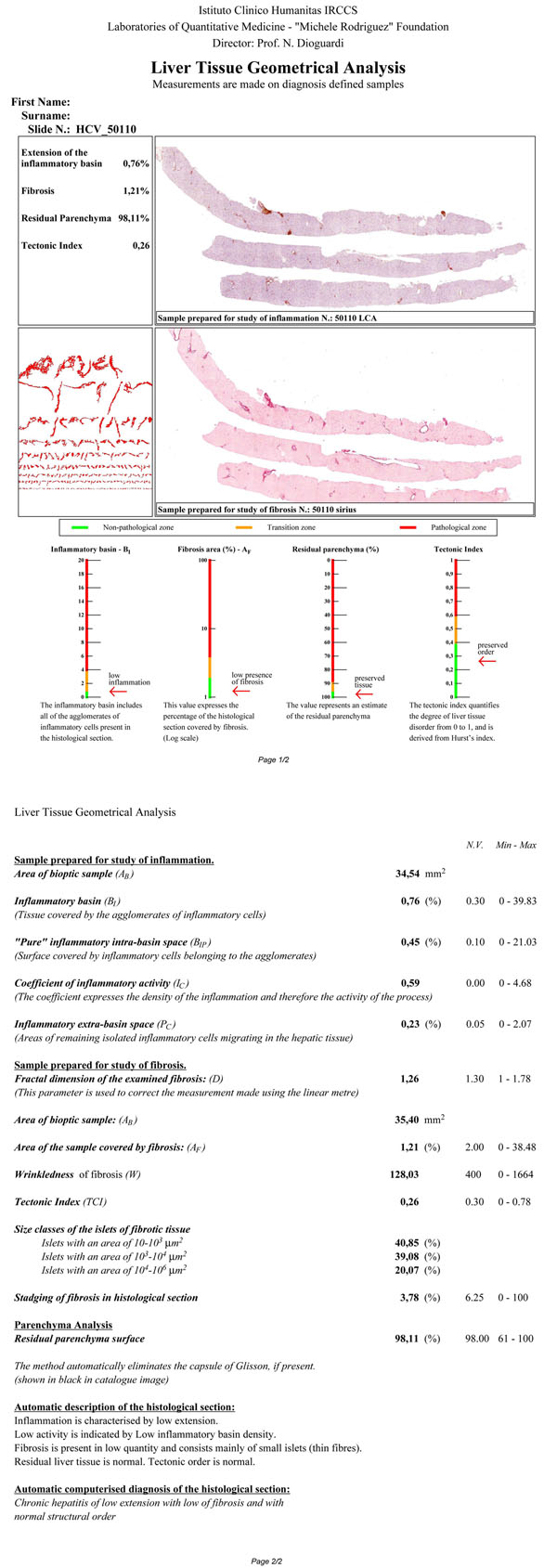
Example of Metrizer output

Furthermore the Metrizer gives: 1) custom image analysis algorithms, 2) objective data obtained from elements present in the tissue, and 3) what can be called an Automatic Diagnosis. This diagnosis, preset for each pathology, is generated by an expert system shaped on a series of data that has been preloaded once diagnosis is known. The expert system referral cut-off is then calculated from these data. The automatic diagnosis permits to completely abolish operator subjectivity, from image capture to the print-out of final report (Figure 2).

### Testing

The validation process of the Metrizer was made by comparing measurements obtained with this machine on histological slides taken from a series of 120 patients previously achieved by the automatic reading software of the preliminary model of automatic analysis, consisting of a motorised microscope Leica DMLA [6].

### Model validation

Complete histological specimen of hepatic biopsies of 235 patients affected by chronic inflammation were acquired, analyzed and compared with clinical data and semiquantitative evaluations. By arranging data and comparing them with the main methods of evaluation in use, cut-offs were located which enabled to opportunely delineate the expert system. From these retrospective cut-offs descriptions of a further 200 patients, making a total of 435 cases analysed with the so-called ‘Model’, were prospectively generated and verified [7].

### Validation of the Metrizer

120 cases coming from the validation series of the Model, which necessitated several months of work on the old system, were then examined in just a few days by the Metrizer and a high level of correlation was reached. Proof of the validity of this new, ultra rapid machine was had by comparing the groups of new data with those obtained by the already validated but much slower semiautomatic Model. In fact the Metrizer carries out all the steps in a few minutes without involving the operator, so that the method which was previously only used for research can be really applied to routine clinical practice in support of the evaluations of the pathologist.

## Results

Comparison of data obtained with the Model with those of new data rapidly achieved by the Metrizer was made on the basis of percentage area values and tectonic index of the fibrosis, and of the inflammatory basin located in the biopsies of the 120 cases available. A double evaluation is thus obtained, from both the old and the new system. The catalogues of located objects in the two different systems were also compared.

The values are very sensitive to intense light and magnifying lens, however by correctly calibrating the machine it is possible to achieve reproducible data with very low margin of instrumental error. The comparison between the two machines can be seen in Table 1. Correlation coefficient between the two sets of data are R=0,94 in fibrosis quantification and R=0,96 in the case of inflammatory basin.

**Table 1:** Model-Metrizer Comparison

	A% Fibrosis		A% Inflammation	
Series	Model	Metrizer	Model	Metrizer
**Min**	0,22%	0,35%	0,02%	0,04%
**Max**	8,32%	14,44%	11,77%	11,48%
**Mean**	2,23%	2,99%	1,87%	1,88%
**St. Dev.**	1,69%	2,33%	2,13%	2,14%

**Relative Median Error (Log):**	9,14%		7,23%	

The table shows that the relative median difference of the calculated values was less than 10%. The highest error can be found comparing cases of fibrosis, which reaches 9,14%. This is due to the diverse composition of the CCD of the Model’s cameras compared with those of the Metrizer, which loses some details in fibrosis. The video camera of the Metrizer have an higher colour definition so as to take even smaller details.

Histological Metrizer (shortened to Metrizer) described herein, is a machine capable of automatically measuring the state of opportunely stained histological specimens in a decidedly more accurate manner than all current methods of evaluation.

The application of quantitative methods in a medical setting faces numerous difficulties because these disciplines involve natural, complex and irregular objects and phenomena, many with characteristics that lead them to be classified as fractal objects [9,10]. Their evaluation describable with traditional linear mathematics and Euclidean geometry, can at times give results that are so distant from reality as to seem caricatures rather than metric descriptions of the object under examination.

However, these objects can be described and, with suitable non-Euclidean geometry, can also be measured with automated calculus technology for operational complexity that requires measurement of their complex structure and interpreted with basics of physics different from those of statics on which the greater part of medical disciplines is founded.

In order to obtain measurements as close as possible to reality, our Laboratory of Quantitative Medicine uses fractal geometry to correct the metric measurements of irregular objects.

## Conclusions

The Metrizer changes the method of examining liver biopsies since it resolves fundamental problems to measure microscopic structures. For the first time it enables evaluation with a scalar number, in an absolutely objective and repeatable manner, the size of the structures that determine the state of the diseased liver. It must be stressed that histological diagnosis of hepatic disease is currently entrusted to the interpretive ability of the pathologist, but it also depends on his level of fatigue.

The automation of the Metrizer standardizes measurement and eliminates operator fatigue, it reads elements which are completely invisible to the human eye and has an unlimited calculation potential.

Furthermore, the Metrizer transforms clinical thought (reasoning) into technological terms and gives both a description and a computer diagnosis, which it obtains using new geometries such as fractals for measuring irregular shapes of scars (collagen islets), fragments of the biliary and the neoangiogenic microvascular network. Being able to measure the real determinants of chronic inflammatory disease dynamics of the liver, means understanding the amount of damage the liver has suffered and refines therapeutic choices, making a more rigorous assessment of its effects.

The totally computerized evaluation entrusted to the Metrizer, i.e. of the liver, completely excludes the subjective intervention of the operator when supplying a “metric hepatogram” where a description is given of the extent of inflammation and fibrosis (scarring), biliary or neoangiogenic regeneration and tissue disorder is quantitatively assessed with a tectonic index. All this is achieved in just a few minutes.

Everyone involved in biologic and medical research will, at some point, come across complex situations which escape thorough description.

All the methods elaborated in the medical world aim at offering adequate procedures with which to locate, define and correlate the largest number of aspects regarding changes in characteristics of the specimen under observation; rather like saying of lesions on organs and organisms determined by natural causes. One way to define tissutal lesions is to study the quality and quantity of the processes causing them and the descriptions of the differences between type, size and intensity of the lesions they cause.

The Metrizer was created to meet the needs of maturity gained in hepatology.

To have measurements which can be repeated anywhere using the same method, means the beginning of a world with less hypotheses and real information.

## List of abbreviations

VM: Virtual Microscope

## Competing interests

No conflict of interest exists.

## Authors' contributions

ND write the manuscript and ideate the theory under the machine and the machine itself, CR revise the text, ideate the software and construct the machine, BF, SDB and SM contribute to revise the text and done the histological preparation, GB revised the text.
